# Compressing the Timelines for Development and Delivery: Accelerating Access to Triple-Drug Therapy to Eliminate Lymphatic Filariasis

**DOI:** 10.4269/ajtmh.21-1174

**Published:** 2022-03-15

**Authors:** Julie Jacobson

**Affiliations:** Bridges to Development, Vashon, Washington

## Abstract

The traditional timeline for a new innovation in public health to move from initial proof of concept to introduction into national programs is sequential and can take decades. Here, we discuss the development of a new drug therapy for lymphatic filariasis (LF) to help progress toward elimination as a public health problem and how this process was accelerated by a group of partners working together. This article documents the way that these partners worked together and made decisions that made it possible to accelerate the process of the development and introduction of the triple-drug therapy involving ivermectin, diethylcarbamazine, and albendazole (IDA). The partners were able to condense the development timeline from the first clinical efficacy data to delivery in a country program for the triple-drug therapy from a projected ∼28 years to less than 5 years while maintaining all of the safety standards. The approach required understanding stakeholders, their roles, need for data to inform decisions, and then looking at timelines focused on prioritizing activities that inform decision-making. This process relied on a close engagement of all stakeholders and good communication. Through this exercise, additional early data review points were added to study designs, studies were run in parallel not sequentially, and a plan put in place to engage all stakeholders necessary for adoption and uptake throughout the process, so they were prepared to make decisions as data became available. This process could provide some insights into how global health can work together in new ways to accelerate the availability of interventions and strategies to promote health and well-being.

## INTRODUCTION

### Background.

Innovation in global public health often follows a predictable pattern. Needs are recognized, ideas are generated, and research is initiated—provided that sufficient resources can be identified. A successful study must then be repeated in a different location, and if the results are replicated, publication will follow and will stimulate discussion of potential practical implications of the findings. If the findings seem important enough, then additional follow-up studies will be required, progressing from an expanded clinical trial that establishes safety and efficacy to an effectiveness trial to see whether the results can be replicated in a real-world setting. At this point, an additional set of supportive data is needed, including data on acceptability, use in different settings, costs, and modeling of impact. Only then will the intervention be considered by the global health community (and the World Health Organization [WHO]) for incorporation into global guidelines.

Because this well-thought-out and deliberate process requires a long timeline, new interventions make it to the field slowly, and people who could benefit from a new intervention often do not receive it for many years (or decades). Any change to speed up this methodical approach requires that partners communicate and interact uncharacteristically—by actually putting their time and resources at risk to accelerate the timeline for programatic implementation and impact.

### Accelerating the standard proof of concept-to-introduction process.

The following discussion documents a successful instance of significant acceleration of the standard proof of concept-to-introduction process. This achievement occurred during the introduction of the triple-drug regimen of ivermectin, diethylcarbamazine, and albendazole (IDA) into WHO’s Global Program to Eliminate Lymphatic Filariasis to replace the annual two-drug treatment of diethylcarbamazine and albendazole (DA) in programs using mass drug administration for elimination of lymphatic filariasis (LF) as a public health problem. The accelerated proof of concept-to-introduction approach and the strategies used during this process are described here, along with the elements that worked well (or not so well). A pathway to such accelerated development is critical to providing ready access to other important advances in global health.

### The acceleration strategy: Understanding the challenge.

#### The lay of the land—who, what, why, and how?

To accelerate any development process, it is useful to start by defining what the targeted end goal is (desired impact), why it is being pursued, and then analyzing the role that the new intervention can play in achieving that goal. It is then essential to recognize who is involved (and who needs to be involved*)* in the decision-making for the intervention to be adopted and scaled up. Only then can the steps to achieving the goal (i.e., the how) be defined and opportunities for acceleration and streamlining be identified.

In the example of IDA, the ultimate target is elimination of LF. If IDA could be introduced safely and effectively, it could accelerate global LF elimination by cutting years off the timeline to achieve this goal. Those ultimately responsible for making the decisions about programs are national-level decision-makers. To inform and support that decision-making, many other actors and partners need to take action by providing the necessary resources (technical, financial, material, policy, and political). To determine how acceleration can be achieved, the critical stakeholders and decision-makers must be identified and their needs for information and other motivating incentives recognized (see Table 1). Each stakeholder has unique perspectives and unique needs, even when aligning to the same final goal. That goal must be defined clearly, the essential role of each partner appreciated, and their decision-making supported by prioritizing data that are aligned to their needs and acknowledging partner incentives that need to be addressed.

**Table 1:** IDA stakeholder analysis

Stakeholder	Asset	Necessary decision	Necessary data	Incentive	Timing
LF-endemic countries	Own and run the programs; provide staff, financial resources, and institutional resources.	If they will adopt a new intervention, plan and execute elements of a new program, train staff, and provide financial and policy-level support.	How will this new intervention help them achieve their goals? Is the strategy recommended by WHO? Do they have sufficient internal or external resources to support the new intervention?	To successfully achieve the elimination program goals, serve their population, succeed in their career, obtain continued or ongoing funding.	Evidence needs to happen early enough to get into planning cycles and go through all the policy and regulatory steps required.
Partners	Provide technical support to country partners, advocate for the program and new initiatives that can help the program succeed.	If the country decision-makers will support the new intervention; if the partner’s donor is aligned with the new interventions; if the intervention is aligned with policy.	Will this intervention help them achieve their project goal and please their donors? Is the intervention feasible and affordable? Will it support their future work in this area?	To achieve their project goals, please their donors, increase impact, generate additional resources, further their institutional mission.	Evidence needs to happen early enough to get into planning and funding cycles and go through all policy and regulatory steps required.
Donors	Financially support program rollout.	If the new intervention aligns with their funding goals and deserves investment; if it is supported by WHO or another regulatory/policy body.	Is the quality, safety, and efficacy of the intervention superior to current treatment?	To align with funding goals and achieve desired public sector impact.	Evidence needs to happen early enough to get into planning and funding cycles and go through all the policy and regulatory steps required.
WHO	Set policies and norms for global health.	If the new therapy is efficacious, and acceptable with supply and cost implications that help achieve the public health goal; if the evidence supports the WHO guidelines development process.	Is the quality, safety, and efficacy of the intervention superior to current treatment? Is the treatment appropriate for the community? Cost? Supply issues?	To achieve the global health objective.	Engagement needs to happen early enough to drive the appropriate data collection for global policy setting and prioritization.
Researchers	Conduct studies to obtain data to support decision-making.	Is this work worthy of publication? Will it support my personal and professional goals?	Is there funding to support the research?	To achieve publications, achieve promotion, be part of achieving a global health goal, continue to receive research funding.	Engagement needs to happen earliest of all, before proof of concept.
Pharma	Provide the drugs needed in the program.	Does this new intervention align with their goals and merit support?	Will the intervention be safe? Will it support corporate social responsibility or other public health goals? Will it support the company’s image and not threaten any revenue streams? Will it create undue risk for the company? What are the cost and investment implications for the company?	To have a positive health benefit, help achieve a global health goal, support a positive image of the company.	Engagement needs to happen early enough to begin data collection to support planning and decision-making, especially related to production issues.

IDA = triple-drug regimen of ivermectin, diethylcarbamazine, and albendazole; LF = lymphatic filariasis; Pharma = pharmaceutical companies; WHO = World Health Organization.

#### The timeline—when?

To appreciate how long the proof of concept-to-introduction process will take and how much acceleration is possible, it is necessary to recognize that each step in the process can move along two types of process times—fixed and variable. Fixed time is the absolute minimum period required to proceed through any single step of the process. For example, the life cycle of the parasite will determine how fast an end point can be measured. Variable time is mostly determined by the human input required to keep the process moving (e.g., discussing concepts, identifying resources, organizing studies, generating agreement, and ensuring political will). Fixed-time steps can be accelerated only by running the fixed segments in parallel and ensuring that data are analyzed for information that will support decision-making as it becomes available. These data may be available prior to completion of a full study but need to be designed into the study and analysis plan from the start to not inadvertently extend the timeline. Appropriate study design, focused on critical data needs can accelerate timelines considerably. Additional opportunity for process acceleration lies either in shortening the periods required for the steps with variable time constraints or skipping those that are not necessary or don’t add value to the decision-making process.

### The initiation and acceleration of IDA for LF through the DOLF project.

On May 6, 2009, a meeting cosponsored by the Global Program for the Elimination of Lymphatic Filariasis and the Bill & Melinda Gates Foundation (Gates Foundation) was hosted at the Task Force for Global Health. The goal was to review current therapies and potential available options for treating LF and onchocerciasis. A key outcome of that meeting was identifying the need to create an initiative that targeted improved treatment tools—optimizing treatment either with current drugs or potentially with repurposed drugs that have already been used in human or animal health.

In June 2010, a grant was made to Washington University in St. Louis, MO—originally titled Optimization of Chemotherapy for Control and Elimination of Onchocerciasis and Lymphatic Filariasis and subsequently renamed the Death to Oncho and LF (DOLF) Project. Its goal was “to develop and test improved treatments for onchocerciasis and LF that will enhance efforts to control and eliminate these important neglected tropical diseases.” The project has since conducted a series of clinical trials administering different combinations, dosages, and frequencies of existing drugs to optimize clinical efficacy. One of these trials was a study of the combined use of IDA in comparison with the standard two-drug regimen of DA. (See article entitled “Lessons from large scale tolerability studies of triple-drug mass drug administration that were performed to support policy change and accelerate elimination of LF” in this Supplement for further information on the DOLF Project. ^1^)

After DOLF Project was funded in June 2010, the IDA project began similar to the other studies, with a technical advisory group that reviewed study plans, then protocols were written, institutional review board approvals were sought, and the study site set up. Ultimately, a small pharmacokinetic (PK) study in Papua New Guinea started in mid-2012 with 24 participants. The three drugs were administered to participants who were observed per the safety protocol for the following 7 days. Samples were taken for the PK measurements and laboratories for 72 hours following the administration of the three drugs. In the third quarter of 2013, the first 12-month efficacy end point was measured.

## RESULTS

### Deciding to accelerate the development timeline.

**Figure 1.**
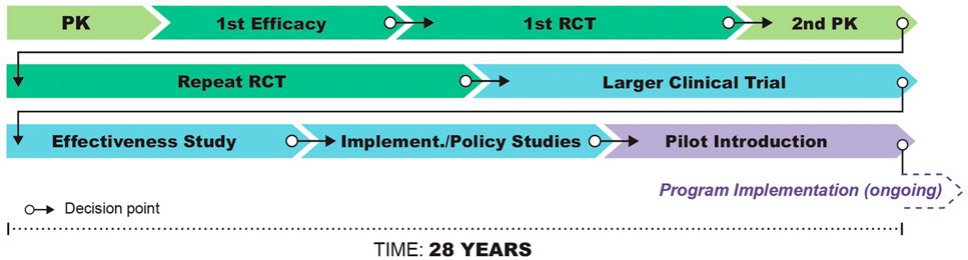
Projected traditional development timeline of ∼28 years for IDA, from proof-of-concept to program implementation. Illustration by Atomic Fox Design.

When the first 12-month efficacy data for IDA were reviewed in 2013, this was the first point at which there was evidence that IDA treatment could be a significant improvement over the standard DA therapy. The results were dramatic. At the American Society for Tropical Medicine and Hygiene meeting in November 2013, which was still more than 2 years before final publication of study results, the investigators met with their program officer to confidentially share the preliminary results and the additional studies being done to confirm with antigen measurement what they had seen by the decreases in microfilaremia. This was the first opportunity to change the timeline in the development of this new regimen by looking at the next steps and how they could be accelerated. This study had a 2-year follow-up period, and results were finally published in February 2016. ^2^ If we project forward to what an illustrative timeline would look like if IDA had continued on this path to development (Figure 1), we can see that the timeline to actual implementation and scale-up for a global program could be very long.

It is a big leap from a 24-person pilot study to implementation at national scale; however, the motivation was to focus on achieving the 2020 elimination goal for LF. It was clear that if this study’s results were replicated, IDA could be a pivotal tool to accelerate progress, but it would have to be available soon. To do this, they would need to assume success and look to disprove the hypothesis early.

At this point, the IDA regimen was moving from the pilot study into the randomized controlled trial (RCT) for efficacy. If the treatment was to be used at scale, there would also need to be data from a trial in a different setting, to reproduce the results of the original RCT that was about to start in Papua New Guinea. In reviewing the project’s study plan, the researchers identified a study in Cote d’Ivoire that was already well advanced in planning, with the study site selected, ethical review underway, and investigators arranged. Adding a study arm to that study, to test the IDA therapy in parallel to the other regimens, was the best opportunity to accelerate the timeline. To align subsequent decision points with the planned Papua New Guinea RCT, a 6-month efficacy readout was added to the Cote d’Ivoire study to look at microfilaremia to see whether the results would replicate what was seen in the Papua New Guinea pilot. This 6-month readout would more closely align to the researchers’ already-planned measurement from the RCT in Papua New Guinea at 12 months.

The investigators also added end points in Cote d’Ivoire that would help them understand the potential mechanism of action 1) by following circulating antigen levels and 2) by using ultrasound investigation of participants identified with worm nests on scrotal ultrasound scans to follow worm viability. This strategy would allow them to follow IDA effects on the adult filarial worms. All of these changes that were required for the Cote d’Ivoire study were discussed with the DOLF Project Technical Advisory Team, and with their support, the IDA protocol was amended, and institutional review board approval sought again. The funder’s project officer participated in the planning and debates and supported a reallocation of grant resources to conduct the new studies, with an understanding that there would be a need for a costed supplement to complete the original project studies if resources were reallocated to Cote d’Ivoire.

All these decisions required good communication and trust between the grantee and the funder and the active engagement of the Technical Advisory Team and country-based research teams.

### Accepting risk—the first acceleration point investment.

Accelerating the timeline introduces risks for stakeholders, including funders, researchers, and grantees:

• There was risk to the funder from investing in a duplication of the primary (post-pilot) study that had not yet been initiated in Papua New Guinea, without any data other than from the small pilot study. This investment in Cote d’Ivoire could be a waste of resources if the study results from the pilot were not replicated in the planned RCT in Papua New Guinea; however, it would take a year to get to the first data on the primary end point and waiting for that data before acting would potentially waste development time. By waiting, they also would have missed the opportunity to build on the planned study in Cote d’Ivoire, which would save months compared with having to plan the study *de novo*.
• The researchers in Cote d’Ivoire carried the risk of adding an arm to an existing study and having to reobtain institutional review board approvals, which could delay the timelines of the original Cote d’Ivoire studies that had been in the planning process for over a year.
• Another substantial risk was to the grantee—the DOLF Project—which had to trust that the reallocated resources would be replaced such that the original studies and objectives would not be compromised. Despite the assurances of the project officer of pursuing the resources and ensuring that funding would be available, no funding commitment can be made until a funder receives a formal grant request, compares it across priorities at the time of submission, and makes a funding decision for award.

### Planning ahead for implementation.

As the RCTs in Papua New Guinea and Cote d’Ivoire were initiated, the project officer began planning for what would need to be done to further accelerate the timeline if the 12-month and 6-month efficacy end points replicated the pilot study findings. First was to think of what the ideal outcome would be if the treatment was determined safe and efficacious. The ideal would be rapid introduction of IDA into national LF elimination programs to speed up timelines for the 2020 elimination goal, especially in places where the programs were anticipated not to reach the 2020 goal—including programs that had not yet started. This prospect would require new WHO recommendations and guidelines, national buy-in for adopting the new treatment, technical partner support, financial support from countries and donors, and available affordable drug supply, per the stakeholder analysis shown in Table 1. The accelerated plan would need to engage all these stakeholders and support their decision-making processes with open communication, as all of the stakeholders would have interdependent roles in potential scale-up of the new treatment. For the IDA development, all of these interdependencies and stakeholder engagement were mapped out and early discussions initiated in preparation for the next set of data that would trigger the following series of decisions.

### The second acceleration decision point.

The next compression point came when researchers collected the first data from the Papua New Guinea RCT 12-month follow-up, which indeed successfully replicated the pilot study results. This positive outcome triggered plans for a multistakeholder meeting to begin brainstorming and planning what would be required to move from early study data to WHO policy and/or national implementation decisions. This relied on and built upon a strong relationship with the WHO to lay out the pathway to implementation and guidelines.

A meeting was held in September 2015, although RCT data were still preliminary and available from only a portion of the participants, to review results and recommend the next steps to get the treatment safely into endemic countries, as appropriate, to accelerate LF elimination. Lymphatic filariasis experts such as the Gates Foundation (Integrated Development, Neglected Tropical Diseases [NTD], and leadership), the DOLF Project study team, WHO, and the Indian national LF program met in Seattle to review the data and the new WHO guideline development procedures for the data required to make recommendations for use. This group made the following decisions:

• Efficacy data from the two ongoing trials (Papua New Guinea and Cote d’Ivoire) would make a sufficient case for the superiority of treatment with IDA.
• The available PK data were not concerning, but additional data would support the submission, as the scale of the proposed program would be up to hundreds of millions of people treated. The PK studies should look at uninfected and infected individuals to differentiate drug effect from treatment effect. Researchers should also ensure sufficient distribution between men and women.
• Safety is the highest priority for treatments used in mass drug administration, which meant a safety database of 10,000 individuals, with good geographic representation and different prevalence of parasite and species, would be needed.
• Diethylcarbamazine could not be considered for use in onchocerciasis- or *Loa loa*–endemic settings without additional studies to show how it could be used safely. As a result, researchers should consider using IDA in these settings only in a second stage of the evaluation, and all current IDA studies would focus on areas endemic for only LF.

Study sites were then selected based on multiple categories that included the previously mentioned points and the significance of the country, from the perspective of regional influence, burden of disease, and potential for providing study data to support national decision-making even before a full WHO recommendation. India and Indonesia were both prioritized as potential early adopters and because of having the largest number of infected people among regions where IDA could potentially be used. Indonesia also offered the opportunity to test the regimen in another filarial species, *Brugia malayi*. Papua New Guinea was prioritized as the most heavily infected area in the world and a natural follow-up to the initial clinical trials. Haiti was identified as providing an opportunity to accelerate regional elimination in the Americas. All countries had links to potential investigators where studies could feasibly be initiated in a timely fashion. The additional PK was done in Cote d’Ivoire, where the clinical trial had been conducted and the research center was prepared to take on additional studies quickly.

The combined studies were designed to mitigate the risk of delays in any of the countries that would hold back a global WHO recommendation. The studies were deliberately overenrolled, and two additional study sites were selected to mitigate the risk of loss of a full site. Sri Lanka was a good opportunity, as the DOLF Project team already had a study site there and the country had some of the most extensive data on LF and transmission parameters, including vector mosquito studies as well as adult and child serology studies. This array of study sites would also provide good data on how IDA might be used in hot spots to complete elimination efforts, and Fiji was proposed to evaluate use of IDA in mop-up efforts as well as for evaluating its impact on scabies, which would be important for the cost-benefit analysis and for acceptability by communities. Ultimately, the project in Sri Lanka did not move forward, and the study in Fiji was conducted later and not included in the first data set provided to support WHO’s guideline development.

Within each of the studies, nested studies were included to address issues that would normally be considered later after RCTs were published and implementation studies complete. The nested studies included information that would support programatic use of the treatment and policy decisions including, acceptability by end users and feasibility studies, which are essential elements in the WHO guideline development process. Beyond clinical development, additional work was started as well, including modeling both for potential impact and supply needs, if the studies were successful, and for different prioritization strategies for IDA rollout. These data were essential to support decision-making at the national level and by the donors who support implementation of national LF programs and make the drugs used in programs.

The modeling work made clear that IDA would not and should not be used everywhere but should be used strategically in targeted areas where it was most needed. The modeling also provided critical data for pharmaceutical (pharma) partners, in particular Merck & Co., Inc., which manufactures and donates ivermectin to support LF elimination. These models helped inform more rational and evidence-based introductory planning with countries and partners and provided clear evidence that IDA would not solve the problems of a weak program with poor implementation. Program strengthening must accompany introduction of the new regimen. Program strengthening to ensure high coverage was important in the discussions with Merck & Co., Inc., as they considered the potential for expanding their drug donation beyond their current commitments for drug supply, already at almost 800 million tablets a year. If they were to increase production, they needed to be assured that the drug would be used properly, not wasted, and not take supply away from where they were already committed.

### Accepting risk—the second investment.

The decision to do all of the implementation field trials with the nested policy studies at the same time carried substantial risk.

• At a minimum, the demands of the multiple trials, all on tight timelines and planned to start simultaneously, were a huge stress on the academic research team. To support the research team, two experienced program managers were hired, each with deep pharma clinical development experience and experience working in low- and middle-income countries. They were embedded into the research team and provided valuable support as a link between the research team and the funder. Resources were also provided to hire a contract research organization for clinical trial support to help with the aggressive timelines in the large clinical trial in India. Study protocols were aligned for easy and relevant study meta-analysis to reach the 10,000-person sample size recommended for safety assessment. Electronic data collection was used so that data could be looked at in real time and issues identified and corrected immediately.
• There was also a risk that an adverse event at one site could threaten the success of the studies in other sites. A data and safety monitoring board and crisis communication plan were put in place in case such an issue arose and to help ensure safety as the studies were initiated and rolled out.
• All of this required significant additional investment. To make these resources available, the president for Global Health from the Gates Foundation had to make trade-off decisions regarding the portfolio. It was a fiscal risk, an opportunity cost, and a potential reputational risk for the Gates Foundation and its staff.
• Merck & Co., Inc. held some of the most significant risk as plans unfolded. Their Mectizan Donation Program is one of the oldest such programs and arguably the one that inspired many of the subsequent NTD donation programs. If IDA proved valuable to the LF program, there would be significant pressure on Merck & Co., Inc. to scale it up. The first locations prioritized for potential use were sites without coendemic onchocerciasis or *Loa loa*, which were currently using DA as their two-drug regimen. This meant that the only additional drug required was ivermectin. However, DA is the regimen used throughout Asia, which is home to the most populous LF elimination programs in the world. If IDA were used everywhere in these countries, it could require up to 800 million new treatments annually. Modeling helped determine how IDA could best be used to have both maximal benefit and feasibility. It was essential that Merck & Co., Inc. be engaged in this process to help them decide whether they could expand their donation and how an expansion could be targeted by the global program. Ensuring that Merck & Co., Inc. had all of the data as soon as it was collected, and that they could query the models as the public health plans were being developed, allowed them to be able to respond at the same time that WHO was preparing their recommendations for the use of IDA.
• The researchers also accepted significant risk in the plan. They agreed to have the data from the trials analyzed by a third party as soon as the sample size reached 10,000 IDA-exposed individuals across the sites. The data were locked and submitted to WHO for independent review before the studies completed enrollment, before the researchers conducted their own analysis, and before any publications were submitted. This separation from their data was a significant risk, which was accepted by the researchers to ensure the data were available for public health decision-making in the timeliest way. Another important and more subtle risk was the amount of time that this accelerated plan consumed. It meant that other projects to which researchers were committed received less time and attention, and this trade-off was substantial.

### Engaging partners and donors.

All the stakeholders shown in Table 1 were engaged throughout the development process. Active engagement included partners from pharma (Merck & Co., Inc., Eisai Co., and GlaxoSmithKline), who were all supporting drugs used in the combination. Most active was Merck & Co., Inc., as the identified use case for IDA would have substantial implications for the demand for ivermectin to be added to current DA regimens, as discussed previously. The major NTD donors supporting LF elimination were also incorporated in outreach; these included the U.S. Agency for International Development, the former United Kingdom Department for International Development, and The END Fund. Program managers and countries were reached through regional program manager meetings and regional program review groups (RPRGs). Partners convened through the Coalition for Operational Research on NTDs, NTD Nongovernmental Organizations Network, International Task Force for Disease Eradication, and Gordon Research Conference, as well as through meetings within and around the American Society for Tropical Medicine and Hygiene. Early time points for sharing data with LF-endemic countries and partners were sought to begin to have national programs consider how IDA could be used within their program setting.

Perhaps, the most critical early engagement was with WHO, which defined the studies that would generate sufficient evidence to inform the WHO guideline development process. World Health Organization has a defined, methodical, and deliberate process for guideline development that is rigorous and takes time. However, the WHO Department of Control of Neglected Tropical Diseases proactively initiated the guideline development process to review IDA and other alternative MDA strategies in parallel to the clinical trials. Crucial to compressing the timeline to implementation was this early start of the process with the active engagement within WHO of the guideline steering committee and guideline development group. Meetings were planned in anticipation of data being available, and literature reviews were initiated so that updated guidelines could be begun and finalized as soon as data became available. ^3^

Particularly important in the partner engagement process was a WHO-convened meeting with broad participation by WHO and other partners, including the Drugs for Neglected Diseases initiative, the Mectizan Donation Program, and the DOLF Project Technical Advisory Team in Geneva on April 5 and 6, 2016. The available studies were reviewed with the full technical input of the Technical Advisory Team and WHO. Important input on adverse event reporting was provided along with clarifications of the requests to Merck & Co., Inc., Eisai Co., and GlaxoSmithKline for drugs for the studies. All the research teams were given specific requirements (with input from the Mectizan Donation Program) for the documentation needed by Merck & Co., Inc. to allow donated drug to be used in the studies; this included the ethical approval protocol, drug request letter, and an importation letter ensuring that ivermectin could come into the country without taxes or fees. An overarching data safety reporting plan was developed for use across all sites. This transparency across partners increased trust and reinforced the interdependent roles of each of the stakeholders.

Early discussion of the implementation and operational research that would need to be done to support the rollout of activities was also an important element. For example, to support the shorter time frame for treatment when using IDA, new stopping criteria would need to be developed. Studies to support the expanded use of IDA in countries with onchocerciasis and *Loa loa* would require specific investments. A research agenda was defined with input from the Coalition for Operational Research on NTDs, the DOLF Project research team, pharma stakeholders, WHO, and other experts. An event/activity timeline is displayed in Table 2.

**Table 2:** IDA timeline

Date	What	Comment
May 6, 2009	“Filariasis Drug Treatment Optimization” meeting, supported by GPELF/Gates Foundation at the LF Support Center Task Force for Global Health	Set up the questions and study ideas for optimization and the need to organize an improved filariasis treatment tools initiative.
June 2010	Funding of DOLF Project	
Mid-2012	Pilot PK study initiated in PNG	
Q3 2013	First 12-month efficacy data available from pilot PK study in PNG	Data indicated higher efficacy.
Q4 2013	American Society for Tropical Medicine and Hygiene discussion between principal investigators and donor program officer	This was the first point acceleration of the timeline was considered. What if this is real? What would we need? Added another country in another region that was a priority for LF for repeat PK and efficacy. Accelerate the planning for the PNG RCT.
August 2014	RCT efficacy study initiated in PNG	
August 2015	First 12-month data available from RCT study in PNG	This data triggered decision making around the second timeline acceleration point
September 2015	Seattle stakeholders planning meeting	
September 2015	Initial IDA data presented at LF AFRO program manager meeting	
November 9, 2015	Briefing of ICMR in India	
February 1, 2016	First publication on IDA data from the pilot study ^2^	If we had waited for this publication, after the 2-year follow-up period, to begin discussing possibilities, we would have added 3 years to the timeline.
March 2016	Initial discussions with Merck & Co., Inc., on studies, potential implications, and ongoing need for engagement	
April 5–6, 2016	DOLF Project Technical Advisory Team	WHO invited and engaged many external stakeholders working on drug development for oncho like DNDi
April 2016	DOLF funding supplement submitted IDA presented at the Gates Foundation WHO team meeting	
May 2016	High-risk districts in India identified where IDA could be targeted	
September 2016	Initial drug forecasting reviewed 24-month efficacy data available from RCT study in PNG	
October 2016	Multicenter IDA safety trial started IDA presentation to AFRO regional program review group	
November 2016	IDA presentation to ITFDE	
December 2016	Presentation on safety of IDA to MDP and MEC	
March 2017	IDA presentation to all staff	
June 2017	Scenarios for IDA introduction developed	
September 26, 2017	India IDA Steering Committee meeting	
September 2017	36-month efficacy data available from RCT study in PNG	
November 2017	Multicenter IDA safety trial completes follow-up WHO guidelines for the use of IDA in LF-endemic countries published ^4^ Merck & Co., Inc. announces an expanded donation to support the introduction of IDA	
December 2017	Final patient follow-up from RCT in PNG; the study is now finished	
2018	Five countries introduce IDA as part of their national LF program	
December 2018	The first RCT in PNG published in the *New England Journal of Medicine* ^5^	Data from the first RCT was published after 5 countries had already introduced IDA into national programs. If the study’s 36-month follow-up data and publication had been used as a decision-making point, this would have added years to the timeline for development and introduction.
May 2019	PK and safety study in CDI published ^6^	
June 2019	Multicenter IDA safety trial published ^7^	

AFRO = World Health Organization Regional Office for Africa; CDI = Cote d’Ivoire; DOLF = Death to Oncho and LF; DNDi = Drugs for Neglected Diseases Initiative; GPELF = Global Program for the Elimination of Lymphatic Filariasis; ICMR = Indian Council of Medical Research; IDA = ivermectin, diethylcarbamazine, and albendazole; ITFDE = International Task Force for Disease Eradication; LF = lymphatic filariasis; MDP = Mectizan Donation Program; PK = pharmacokinetic; PNG = Papua New Guinea; RCT = randomized controlled trial; WHO = World Health Organization.

In addition to research team meetings with the field sites, weekly team meetings were held with the research team, the project officer at the donor, and the two supporting consultants. The President for Global Health led biweekly meetings with a Gates Foundation leadership team to review progress and address concerns and issues; this team included members across the Office of the President and the Integrated Development and NTD teams. They provided strategic input and helped facilitate bringing in additional resources to support the work. An IDA Triple-Drug Investment team within the Gates Foundation was also established and met monthly to review progress with a broader team and address any arising issues.

### Outcome.

**Figure 2.**
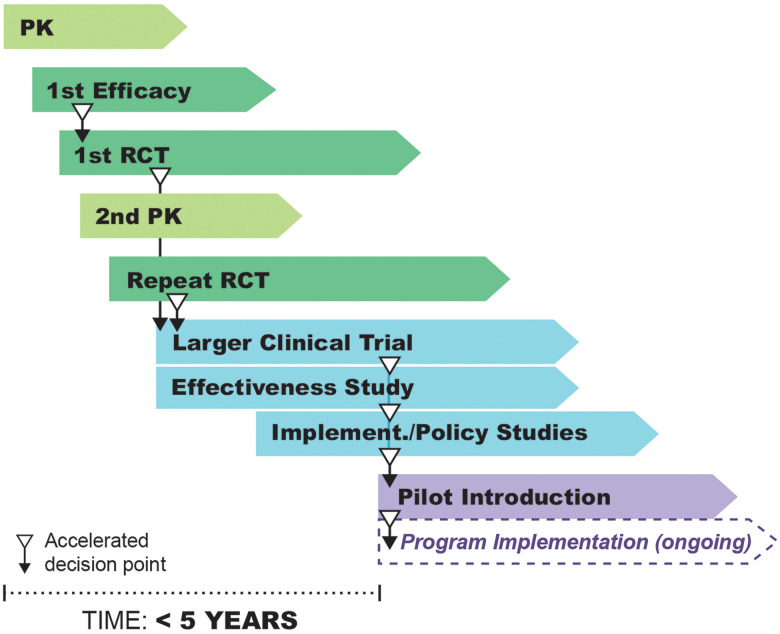
Accelerated timeline of under 5 years for IDA from proof-of-concept to program implementation, showing points where timelines were accelerated. Illustration by Atomic Fox Design.

The outcome of this collective work was remarkable. When WHO released their guideline with recommendations on the use of IDA for LF elimination, ^4^ Merck & Co., Inc. was able to announce an extension of their donation immediately following, and early adopter national programs were aligned with their donors (predominantly the END Fund) and partners to rapidly take up the new treatment into their programs as seen in the timeline of some activities in Table 2. The IDA triple-drug therapy was ultimately introduced into five country programs before the first RCT was published. If we consider the start of the timeline from the first efficacy data available prior to the publication of the first paper (from the pilot study), this compressed an anticipated timeline of 28 years to less than 5 years (Figure 2). This work demonstrated how effective a consciously proactive approach could be for product development when focusing on a specific goal and working collaboratively across all stakeholders. ^1^

## DISCUSSION

**Figure 3.**
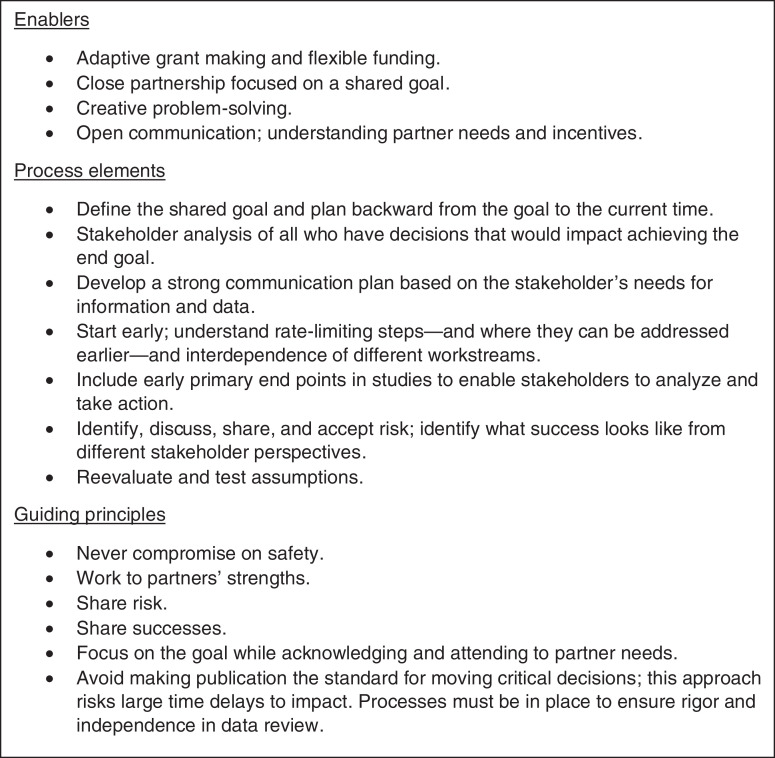
Enablers, process elements, and guiding principles of the accelerated process.

Several factors emerged from this experience, fitting into three broad categories—enablers, guiding principles, and process elements (Figure 3). Enabling factors included the close partnership, with each stakeholder being committed to the program goal and willing to do their part to help the new intervention move forward. This partnership was also based on a core of agreed principles, some of which were openly acknowledged, like the commitment to safety without compromise, whereas others were more subtle, such as working to each partner’s strengths and acknowledging partners’ different needs.

From a process standpoint, certain steps were required to create the overall plan. The earlier you start, the more potential for compressing the timeline; however, an early start also means accepting increased risks. It is important to identify critical milestones and timepoints to reevaluate whether you are on the right path or whether additional tasks need to be completed that were not anticipated. In the IDA development, the stakeholder analysis was an important element for understanding who should be engaged when and how. The first step always remains to think backward from the goal and plan forward to the steps it will take to get there in a responsible and thoughtful way. Every step and action should align with the ultimate goal and decision-making as soon as the data required are available. Interesting science that is not part of the critical steps to the goal can distract and slow progress. Clarifying the goal can allow the prioritization and streamlining of activities to focus on the most judicious path.

With active planning and strong partnership, it is possible to responsibly condense timelines in the development and introduction of new interventions. Each program is unique, but the general principles and processes set out in Figure 3 should be applicable to other development and introduction efforts to increase impact, improve health, and save lives.

Across the articles in this Supplement, we provide more detail about the experience of the development and rollout of the new IDA treatment from different stakeholders’ perspectives. Ivermectin, diethylcarbamazine, and albendazole combination was unique in many ways:

• First, all the drugs in the combination have a long history of use in programs with extensive safety data; scaled-up programs exist that IDA could be integrated into; and drug donation partners are already making high-quality products at scale.
• The Gates Foundation, as the donor, was willing to adapt their grant making process, accept financial risk, and use pharma experience to efficiently “get to market”—and invested accordingly.
• The LF community already had strong partnerships and a well-established goal, with national buy-in and strong leadership from WHO.

All these elements helped accelerate the timeline from clinical trials to national program implementation. These elements also make this situation unique and not necessarily generalizable. Therefore, we will also explore in this Supplement examples from different areas—such as diagnostics, vaccines, and novel drug development—to see what common elements we can define and use to think differently about how innovations make it to people in need. It should be a challenge to all of us who work in global health—donors, researchers, program managers, pharma, or endemic country decision-makers, to work together more efficiently to achieve better health for the people we serve.
